# Supplementary material for: CDK9-dependent RNA polymerase II pausing controls transcription initiation
Source: eLife. 2017 Oct 10;6:e29736. doi: 10.7554/eLife.29736 (PMC5669633; doi:10.7554/eLife.29736)
Supplement: Supplementary file 1. — Note that the conclusions we draw across different cell-lines are all based on metagene analysis, involving from 500 up to more than 2000 genes. Thus, we assume cell-line specific differences to have an insignificant influence and that the tendencies we observe rather suggest strong conservation. [file elife-29736-supp1.docx]

**Supplementary File 1.** **Published datasets used for analysis.**

Note that the conclusions we draw across different cell-lines are all based on metagene analysis, involving from 500 up to more than 2000 genes. Thus, we assume cell-line specific differences to have an insignificant influence and that the tendencies we observe rather suggest strong conservation.

| **Experiment** | **Factor** | **Cell type** | **GEO ID** | **Source** |
| --- | --- | --- | --- | --- |
| FAIRE-seq |  | K562 | GSE35239 | Lieb (Consortium, 2012) |
| DNase Hi-C |  | K562 | GSE56869 | Duan (Ma et al., 2015) |
| Bisulfite-seq |  | K562 | GSE27584 | Myers (Consortium, 2012) |
| ChIP-seq | TOP1 | HCT116 | GSE57628 | Levens (Baranello et al., 2016) |
| DNase-seq |  | K562 | GSE32970 | Crawford (Consortium, 2012) |
| DMS-seq |  | K562 | GSE45803 | Weissman (Rouskin et al., 2014) |
| DNase-seq |  | K562 | GSE29692 | Stamatoyannopoulous (Consortium, 2012) |
| ChIP-seq | NELF-E | K562 | GSE31477 | Struhl (Consortium, 2012) |
| TOP1-seq |  | HCT116 | GSE57628 | Levens (Baranello et al., 2016) |
| ChIP-seq | Pol II (S2P) | Raji B | GSE52914 | Andrau (Descostes et al., 2014) |
| ChIP-seq | Pol II (S5P) | Raji B | GSE52914 | Andrau (Descostes et al., 2014) |
| ChIP-seq | Pol II (S7P) | Raji B | GSE52914 | Andrau (Descostes et al., 2014) |
| ChIP-seq | Pol II (Y1P) | Raji B | GSE52914 | Andrau (Descostes et al., 2014) |
| ChIP-seq | Pol II (Y1P) | Raji B | GSE52914 | Andrau (Descostes et al., 2014) |
| MNase-seq |  | Raji B | GSE52914 | Andrau (Descostes et al., 2014) |
| ChIP-seq | CDK9 | HEK293T | GSE51633 | Rosenfeld (Liu et al., 2013) |
| ChIP-seq | CDK9 | HCT116 | GSE70408 | Shilatifard (Chen et al., 2015) |
| ChIP-seq | Brd4 | HEK293T | GSE51633 | Rosenfeld (Liu et al., 2013) |
| ChIP-seq | Brd4 | HeLa | GSE51633 | Rosenfeld (Liu et al., 2013) |
